# Accurate Measurement of the Optical Properties of Single Aerosol Particles Using Cavity Ring-Down Spectroscopy

**DOI:** 10.1021/acs.jpca.2c01246

**Published:** 2022-04-25

**Authors:** M. I. Cotterell, J. W. Knight, J. P. Reid, A. J. Orr-Ewing

**Affiliations:** † School of Chemistry, University of Bristol, Cantock’s Close, Bristol BS8 1TS, U.K.

## Abstract

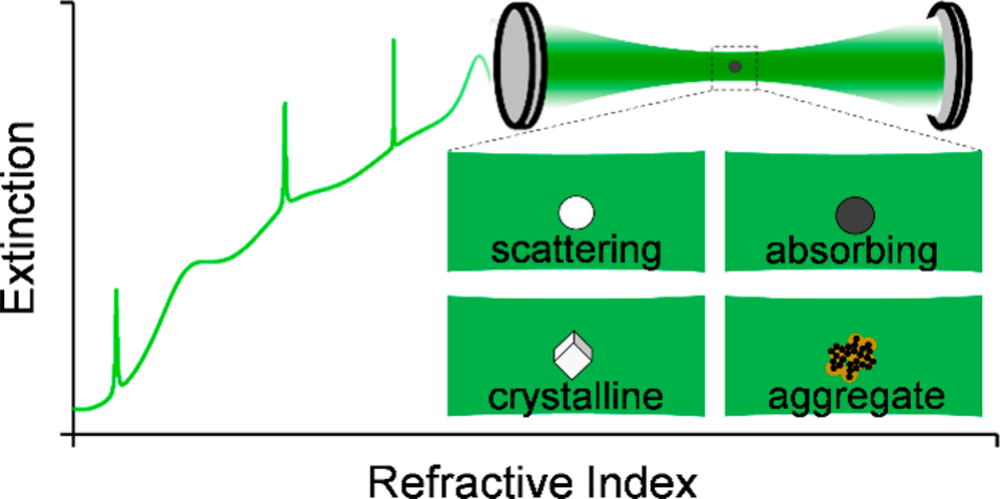

New approaches for the sensitive and accurate quantification of aerosol optical properties are needed to improve the current understanding of the unique physical chemistry of airborne particles and to explore their roles in fields as diverse as chemical manufacturing, healthcare, and atmospheric science. We have pioneered the use of cavity ring-down spectroscopy (CRDS), with concurrent angularly resolved elastic light scattering measurements, to interrogate the optical properties of single aerosol particles levitated in optical and electrodynamic traps. This approach enables the robust quantification of optical properties such as extinction cross sections for individual particles of known size. Our measurements can now distinguish the scattering and absorption contributions to the overall light extinction, from which the real and imaginary components of the complex refractive indices can be retrieved and linked to chemical composition. In this Feature Article, we show that this innovative measurement platform enables accurate and precise optical measurements for spherical and nonspherical particles, whether nonabsorbing or absorbing at the CRDS probe wavelength. We discuss the current limitations of our approach and the key challenges in physical and atmospheric chemistry that can now be addressed by CRDS measurements for single aerosol particles levitated in controlled environments.

## 1 Introduction

Although much of the growing public discussion of the threats of global warming focuses on greenhouse gas levels in the atmosphere and future net-zero CO_2_ emission targets, atmospheric aerosol particles also contribute significantly to the radiative balance that determines Earth’s climate.^1−4^ Atmospheric aerosol particles have a variety of natural and anthropogenic sources, from wind-blown mineral dust^5^ and biomass burning^6−8^ to condensation of oxidized organic compounds emitted by vegetation, transportation, and industrial processes.^9,10^ These nanometer to micron-scale particles are dispersed throughout the Earth’s troposphere, where they scatter and absorb incoming solar and outgoing terrestrial radiation. In addition, tropospheric aerosols affect cloud formation, brightness, and lifetime by nucleating the growth of water droplets. These processes are known as the aerosol *direct* and *indirect* effects, respectively, and both influence the reflectivity (albedo) and absorptivity of the atmosphere to solar and terrestrial radiation. Greater cloud cover and an increase in nonabsorbing aerosols such as sulfuric acid particles have a net cooling effect on the atmosphere by reflecting sunlight back into space. Research efforts in geo-engineering seek to exploit this cooling by deliberate injection of fabricated particles into the stratosphere to mitigate some of the climate impacts of rising tropospheric CO_2_ concentrations.^11^ In contrast, absorbing aerosol particles trap the solar radiation and release its energy as heat to the atmosphere. Important examples of absorbing aerosol particles are black carbon (BC, e.g., soot particles produced by combustion processes in industry and transportation as well as from wildfire sources undergoing flaming combustion)^7^ and brown carbon (BrC, e.g., from the smoldering and pyrolysis phases of biomass combustion, whether from wildfires or agricultural practices).^12^

Research efforts to quantify the contributions of greenhouse gases and aerosols to the radiative balance in the atmosphere (quantified by the *radiative forcing*) are summarized in recent Intergovernmental Panel on Climate Change (IPCC) reports.^1,2^ These reports assess the range of global and regional temperature rises predicted by atmospheric models for prescribed emissions and remediation scenarios. At present, some of the largest uncertainties in the IPCC tabulations of radiative forcing derive from the direct and indirect contributions of atmospheric aerosols. These uncertainties stem in large part from poor quantification of how aerosols scatter and absorb electromagnetic (EM) radiation. While improved observations of aerosol optical properties from atmospheric research aircraft and remote sensing (whether ground-based or satellite-borne) platforms provide valuable data, a fundamental understanding of how aerosol properties evolve with atmospheric processing and with photochemical aging is crucial in improving predictions of aerosol–light interactions over the particle lifetimes.

Consider, for example, a biomass burning plume. Large concentrations of soot (comprising aggregates of carbon spherules) are emitted, in addition to semivolatile organic species and water vapor. In the initial ∼1 h following emission, condensation of the gaseous emitted species onto the soot particles causes the soot aggregates to restructure, thus changing the intrinsic absorption and scattering cross sections of the soot component. In addition, a coating of water and organic compounds can act as a lens, focusing light onto the coated soot particle, and therefore enhances the amount of light absorbed by the particle.^13,14^ Over the remaining 7–10 days of the particle’s lifetime, the coatings undergo chemical and physical transformations, caused by photobleaching and oxidative bleaching of molecular chromophores and gas-to-particle partitioning of further semivolatile or low-volatility organic species. These processes change the intrinsic optical properties of the coating material as well as the magnitude of the lensing effect.^8^ For different types of aerosol particles undergoing such transformations, accurate characterizations of the changing optical properties and the associated kinetics are crucial for an improved understanding of how aerosols impact atmospheric composition and climate. These evolving properties cannot be inferred from laboratory studies of bulk chemical systems because the unique physicochemical properties of nanometer to micron-scale particles promote different chemistries and reaction rates,^15^ necessitating measurements using well-defined aerosol samples.

A better understanding of how atmospheric aerosol particles undergoing continuous photochemical and physical processing interact with solar radiation and how these aerosol particles seed cloud formation is therefore a priority in atmospheric chemistry and climate science. Here, we focus on methods for accurate and precise determination of the optical properties of aerosol particles that control how they scatter and absorb sunlight. New capabilities to measure these evolving optical properties will provide a gateway to future studies of the kinetics of chemical change in atmospheric aerosol particles under controlled laboratory conditions.

Although the focus here is primarily on atmospheric aerosols, the benefits of accurate measurements of changes in aerosol scattering and absorption should also extend to other applications of aerosol science, for example in chemical synthesis and healthcare. Recent demonstrations of accelerated reactions in micron-scale droplets offer new avenues for sustainable chemical synthesis, but the reasons for significantly enhanced reaction rates remain the subject of intensive investigation.^16−18^ A newfound ability to monitoring changes in chemical composition of micron-scale single aerosol particles using absorption spectroscopy may help to unravel these causes. In healthcare applications, the optical properties of pathogen-containing droplets may influence pathogen survival rates upon droplet exposure to UV germicidal radiation.^19,20^ UV absorption measurements of such droplets could contribute to the development of improved sterilization technologies. Progress toward these and other impacts requires new spectroscopic tools that can track changes in composition using measurements of light absorption to unravel the fundamental chemical, physical, and biological processes at work in aerosol particles.

Regardless of the field of research, quantitative study of the optical properties of aerosols presents many challenges, for both measurements and the interpretation of the resulting data. Whether in the natural environment, an aerosol reaction chamber that simulates atmospheric processing,^21^ or droplets in exhaled breath, the aerosol particles present are typically heterogeneous in size, shape, chemical composition, and constituent phases. Although aqueous aerosol microdroplets are spherical, they may contain inclusions or transform to nonspherical particles upon drying and efflorescence. Moreover, solid particles adopt a wide range of morphologies from crystalline to nanostructured or amorphous structures with shapes that depend on chemical composition and the rate of particle drying. The scattering and absorption properties of the aerosols depend critically on their shapes, not least because the dimensions of the particles are similar to the wavelengths of visible, near-infrared, and ultraviolet light that might be used to probe spectroscopically their composition or that they might interact with in the troposphere. The outcomes of any measurements of the propensity for light to scatter and be absorbed upon interaction with a particle, quantified by the *single scattering albedo* and *coalbedo*, will therefore be highly sample dependent.

Even for well-defined spherical droplets of single-component liquids or binary solutions prepared under controlled laboratory conditions, quantitative spectroscopic studies are difficult because an aerosol sample comprising numerous particles is typically needed to obtain measurable changes in light extinction (corresponding to the sum of scattering and absorption contributions).^22−30^ In such aerosol *ensemble* measurements, the particles have a distribution of sizes, and the number of particles must be well characterized, just as the partial pressure of a gas sample must be known for Beer–Lambert law measurements of molecular absorption cross sections. These problems can be largely addressed in the laboratory by use of devices such as a differential mobility analyzer (DMA) and condensation particle counter (CPC) that select a narrow mobility size distribution of aerosol particles prior to spectroscopic characterization and that count the number of particles at the exhaust of the optical spectrometer, respectively. Nevertheless, difficulties remain in making the highly precise and accurate measurements of optical parameters needed for atmospheric modeling because the size distributions of DMA-classified particles are not monodisperse, or even monomodal, and are subject to large uncertainties. Moreover, the number concentrations reported by CPCs can be biased by ±10% depending on their calibration accuracy, and the numbers of particles intersected by the probe light fluctuate over the measurement time scales.^26,27,30−34^

A different strategy is instead to make measurements of scattering and absorption for single particles. This approach is one we have advocated for superior accuracy and precision in determination of aerosol optical properties, but it presents its own challenges to implement. Sensitive spectroscopic techniques are required to determine the extinction of incident light caused by a single particle only a few microns or less in diameter, and the particle size, shape, and structure (phase) must be well determined for a meaningful interpretation of the measurements. Our solution to these challenges combines the ultrasensitive technique of cavity ring-down spectroscopy (CRDS) with confinement of a single particle in an optical or electrodynamic trap over extended time scales.^35−42^ CRDS measures absolute particle scattering and absorption losses directly, and a sequence of such measurements for a trapped particle evolving in size can be analyzed to extract the corresponding real and imaginary components of the refractive index, both of which are indicative of chemical composition and are parameters required for atmospheric radiative transfer calculations. Such an analysis involves comparing the sequence of CRDS-measured extinctions to predictions from an optical model that best represents the interaction of the particles with light (such as Lorenz–Mie theory for particles known to be homogeneous spheres). The precision and accuracy of refractive index determinations from single-particle CRDS data are sufficient to reduce uncertainties in predictions of the aerosol direct effect on radiative forcing.^39,43^

Alternative approaches use nephelometry to measure aerosol scattering^44^ or photoacoustic spectroscopy (PAS)^32,45−49^ to quantify absorption for aerosol ensembles, with both techniques now extended to measurements on single, levitated particles.^50−53^ However, PAS requires calibration to convert measured signals to absorption cross sections,^54−56^ and it encounters quantification problems for particles containing volatile components.^51,52,57^ Nephelometry also requires careful calibration with a known aerosol standard, and measurements suffer from biases arising from the requirement to truncate the angular range over which scattered light is collected, which vary substantially with aerosol absorption strength and particle shape.^58^ Other innovative and recently developed single-particle spectroscopy approaches include analysis of the light scattered from a broadband light source to obtain particle size and wavelength-dependent refractive index information,^59−62^ and photophoretic spectroscopy of weakly absorbing aerosol particles levitated in a double-ring electro-dynamic balance.^63^ Aerosol optical properties can also be determined by monitoring the size changes induced by laser heating of weakly absorbing droplets held in optical traps.^41,64,65^ This contribution will focus on CRDS methods, and it will illustrate recent developments in the determination of both the scattering and absorption contributions to the extinction for single aerosol particles as small as 1 μm in diameter. We discuss the utility of these methods for studies of spherical and nonspherical particles, whether nonabsorbing (i.e., scattering-only) or absorbing, and their potential to address challenges in atmospheric science and other fields.

## 2 Optical Properties of Aerosol Particles

Aerosol particles in the atmosphere can reflect, refract, diffract, and absorb radiation, thereby influencing the radiative forcing of the atmosphere by light scattering and absorption. The degree to which they scatter and absorb different wavelengths of light depends on the shape, size, composition, and structure of the particles. Of particular interest here, the chemical composition is directly connected to the complex refractive index (*m*), which has real (*n*) and imaginary (*k*) parts, *m* = *n* + *ik*. The speed of light in the particle is determined by the real component, whereas the imaginary component governs the attenuation of the light by absorption, with the two components linked by the Kramers–Kronig relationship.^66^ Both *n* and *k* depend on the wavelength of the light, and this dependence is referred to as the dispersion. For spherical and homogeneous particles, the scattering and absorption of EM radiation by particles are well-described by Lorenz–Mie theory. The scattering and absorption cross sections are respectively defined as  and , where *I* is the incident irradiance and *W*_sca_ and *W*_abs_ are the scattered and absorbed power. The overall extinction cross section is σ_ext_ = σ_sca_ + σ_abs_ and is related to the geometric cross section of the particle (σ_geom_) by the extinction efficiency *Q*_ext_:
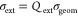
1Similarly, the scattering and absorption efficiencies (*Q*_sca_ and *Q*_abs_) connect the scattering and absorption cross sections to the geometric cross section of the particle, and their sum is *Q*_sca_ + *Q*_abs_ = *Q*_ext_.

The ratio of scattering to total extinction is known as the single scattering albedo (ω = *Q*_sca_/*Q*_ext_), which quantifies the fraction of light extinction caused by scattering losses. The fraction of incident light lost by absorption is then given by the coalbedo, 1 – ω. The single scattering albedo is central to the assessment of contributions from different types of particles to the aerosol direct effect on the radiative forcing of the atmosphere.^3,4^ Optical measurements of the loss in intensity of light transmitted through an aerosol sample typically measure the total extinction. Hence, separating scattering and absorption losses represents a challenge that must be overcome to understand how aerosols affect the climate.

**Figure 1:**
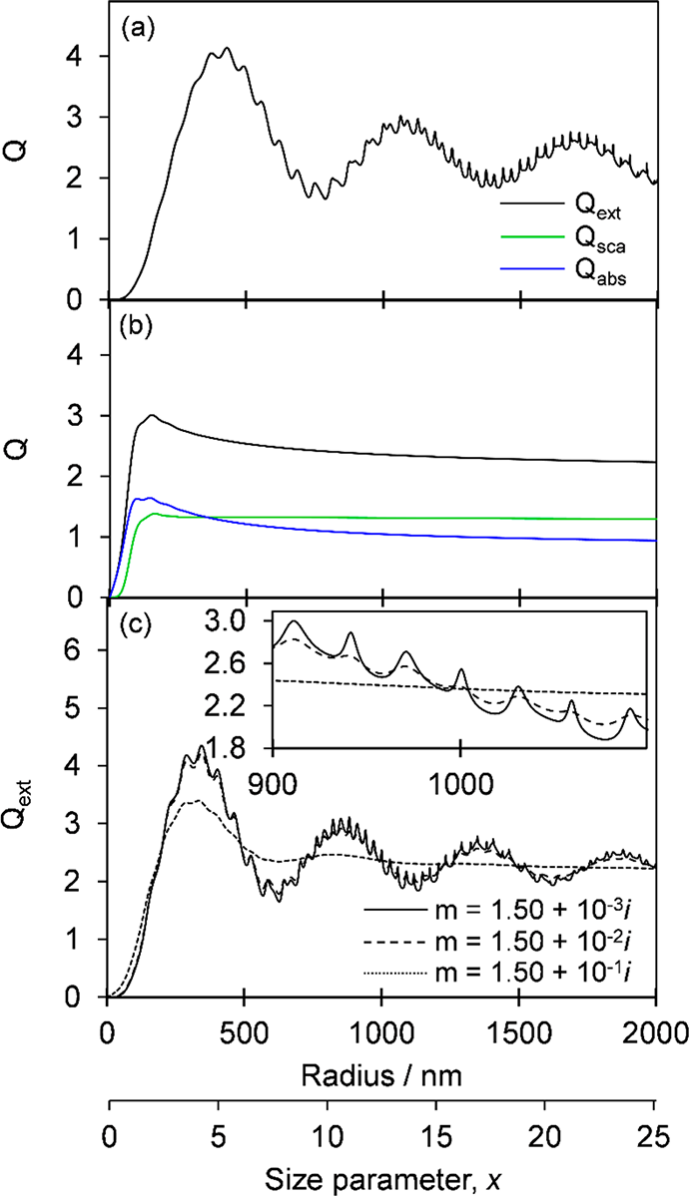
Scattering, absorption, and extinction efficiency calculations from Mie theory for (a) a nonabsorbing droplet with *m* = 1.40 + 0.00i, with scattering dominating the extinction; (b) an absorbing sphere with a refractive index representative of BC (*m* = 1.95 + 0.79i); and (c) absorbing spheres with *n* = 1.50 but with progressively increasing *k* from 10^–3^ to 10^–1^. The wavelength used in these simulations is λ = 500 nm. All data are plotted as a function of particle radius (*r*) and the corresponding scale for size parameter (*x* = 2π*r*/λ) is also shown.

The measurements reported in this Feature Article illustrate that the scattering and absorption cross sections can be separately quantified, although the methods described have only been applied so far to particles that are homogeneously mixed and single phase. Some aerosol particles of atmospheric interest are inhomogeneous and contain more than one phase, such as those with core–shell (i.e., coated sphere) morphologies. Core–shell particles should be amenable to study using our methods because modifications to the Lorenz–Mie equations successfully describe the optical properties for such structures.^66^ To illustrate the principles behind our approach, consider two examples of homogeneous spherical particles of radius 200 nm exposed to 500 nm wavelength sunlight, one of which is scattering but nonabsorbing and the other of which is strongly absorbing. A droplet comprising an aqueous solution of NaCl (which may represent a sea-spray droplet) is nonabsorbing and so has a single scattering albedo ω = 1.0 and coalbedo of 0.0, whereas an absorbing particle with the same size and a refractive index for BC (soot) has ω = 0.47 and hence a coalbedo of 0.53. We note that soot particles are aggregate structures and their optical properties are not represented well by a spherical model, but we compare these two examples of an aqueous NaCl droplet (*m* = 1.40 + 0.00i, a value appropriate for such a droplet suspended in air with a relative humidity (RH) ≈65% and for green light^40^) and an absorbing sphere (*m* = 1.95 + 0.79i for the BC analogue particle^67^) to illustrate the broad impacts of the real and imaginary components of the complex refractive index on optical parameters. For two such particle types, Figure 1 shows plots of the dependence of *Q*_ext_ on particle size, also expressed as a dimensionless size parameter *x* = 2*πr*/λ, where *r* is the particle radius and λ is the wavelength of incident light. Such plots of the variation of *Q* with size parameter are common, but this representation can be misleading because it suggests that the wavelength dependence of *Q* is known for a fixed particle size, requiring knowledge of the dispersions in *n* and *k*. The plots also show the *Q*_sca_ and *Q*_abs_ contributions to *Q*_ext_.

The broad oscillatory structure in these plots arises from interference between light waves passing around and through the particle. The superimposed sharp resonance structure arises from light coupling into resonant modes of the spherical particle that trap the light by total internal reflection. At specific resonant frequencies, the trapped light undergoes numerous reflections within the droplet, which behaves as a spherical resonator with a high-quality factor, and the propagating EM waves interfere constructively.^66^ The resulting modes are commonly referred to as Whispering Gallery Modes (WGMs) or Morphology Dependent Resonances and, together with the interference structure, provide a fingerprint of particle size and refractive index. Hence, they have been exploited in particle sizing, refractive index determination, and stimulated Raman scattering studies of aerosol composition by precise comparison of WGM resonance frequencies with predictions from Lorenz–Mie theory.^68,69^ Light attenuation by absorption in the case of the soot particle reduces the quality factor of the spherical resonator and so broadens and reduces the amplitude of the resonances to the point where they are not resolved in strongly absorbing particles. This phenomenon is emphasized in further calculations presented in Figure 1c, showing the progressive damping of the resonance and interference structures in *Q*_ext_ for spherical particles with *n* = 1.50 as *k* increases from 10^–3^ to 10^–1^. Nonsphericity of the particles also broadens or quenches entirely these structures. The measurement at high resolution of such interference and resonance structure in extinction cross sections is key to our methods of determination of the real and imaginary refractive index components from CRDS measurements of single, trapped aerosol droplets described in this article. Efforts to make such CRDS measurements for flowing ensembles of size-selected aerosol particles suffer from loss of the fine resonance structure because of the inevitable polydispersity in particle size and the Poisson statistics of fluctuating numbers of particles in the intracavity laser beam volume.^26,27,30−34^

## 3 Single-Particle CRDS Methods

Measurements of the extinction of light by a single aerosol particle only a few microns in diameter require a combination of three key experimental components: (i) a method to trap the particle in a small region of space over extended time durations; (ii) a highly sensitive spectroscopic method to measure the weak extinction by such a small particle; (iii) control of the environment conditions of temperature, relative humidity, and dust-free gas flow around the particle. We have met all these requirements by combining a sample cell housing a Bessel laser beam (BB) optical trap for nonabsorbing, spherical particles or a linear electrodynamic quadrupole (LEQ) trap for particles of any absorption strength and shape with a continuous wave (cw) CRDS spectrometer. The spatial confinement of the particle by the BB or LEQ trap must be sufficient to hold the particle at the central maximum of a TEM_00_ Gaussian mode of the ring-down cavity, which has a beam waist under our experimental conditions in the range 250–300 μm.^36,37,39^ This degree of confinement is necessary because the measured extinction depends on the radial displacement of the particle from the center of the TEM_00_ cavity mode.^35,70^

The use of cw CRDS with a single-mode cw excitation laser and a high-finesse optical cavity has several advantages over pulsed laser CRDS (for example, using nanosecond pulsed laser sources) particularly when interrogating single particles. Most important is that the longitudinal and transverse mode resonance frequency conditions of the optical cavity can be used to ensure that a single TEM_00_ cavity mode with well-defined spatial profile is exclusively excited. Excitation of non-TEM_00_ cavity modes and the confounding impacts these could have in measurements of the extinction cross section for a trapped particle can be distinguished from the interaction of the particle with TEM_00_ cavity modes only when using cw CRDS. A further advantage comes from the typically higher sensitivities that can be achieved from cw CRDS with a well-aligned cavity, allowing measurements on single particles below 1 μm in diameter.

The operating principles and design considerations for a cw CRDS spectrometer are discussed elsewhere.^71−73^ Briefly, our cw CRDS instruments use single-frequency diode laser sources with line widths <5 MHz, emitting at visible wavelengths of either 405 or 532 nm. The laser beam passes through an acousto-optic modulator, from which the first-order diffraction beam is selected and focused into a high-finesse linear optical cavity. This cavity consists of two concave mirrors with high reflectivity (*R* > 99.99%) at the frequency of the laser source, separated by a fixed length of 0.5 or 0.8 m for the 532 and 405 nm spectrometers, respectively. One of the mirrors is affixed to a piezo-ring transducer that constantly varies the mirror-to-mirror separation over several micrometers. This cavity length modulation is necessary to tune the cavity into resonance with the laser frequency; light is sustained within the cavity when the so-called *standing wave* condition is fulfilled such that the cavity length is equal to an integer number of wavelengths of the laser light. The intracavity beam then undergoes multiple reflections between the two mirror surfaces and constructively interferes such that light intensity builds up (or *rings up*) inside the cavity. A small fraction of this light leaks out of the cavity mirrors and is detected by a photodiode. The output photodiode voltage is directly proportional to the intracavity light intensity. When this voltage reaches a chosen threshold value, the acousto-optic modulator is triggered to extinguish the first-order diffraction beam. Subsequently, the intracavity light intensity decays (*rings down*) exponentially. As we discuss below, the characteristic exponential time constants for these decays are used to measure directly single-particle extinction cross sections. Most of the cavity is purged with nitrogen gas to ensure dust and gaseous absorbers do not contribute to changes in the CRDS-measured extinction.

**Figure 2:**
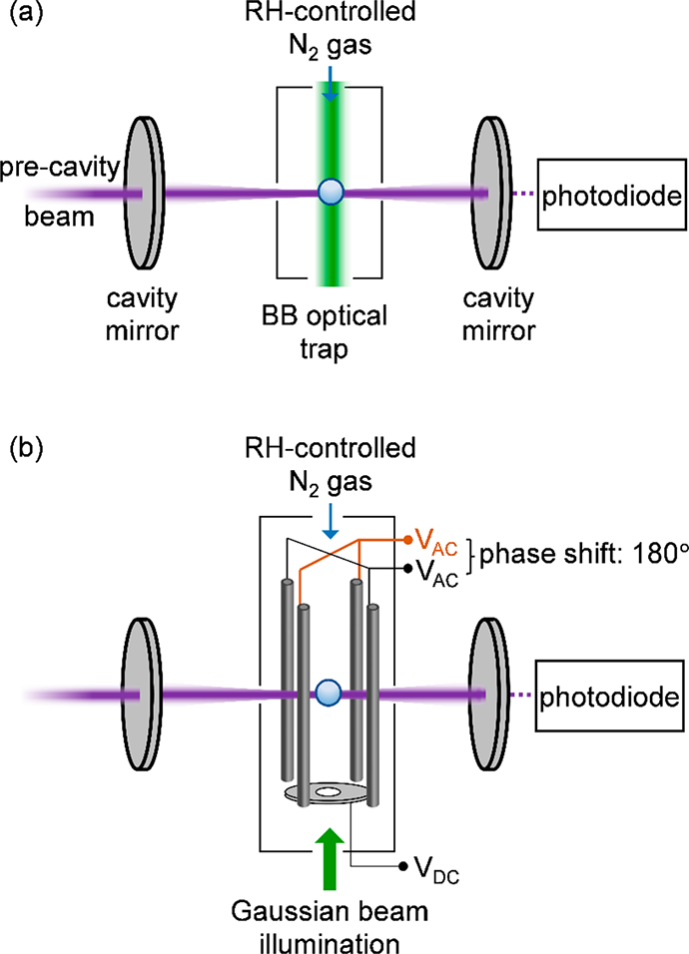
Schematic diagrams for the cw CRDS interrogation of a single aerosol particle confined using (a) a Bessel laser beam optical trap or (b) a linear electrodynamic quadrupole trap. *V*_AC_ and *V*_DC_ denote applied AC and DC voltages, respectively.

In our experimental designs illustrated in Figure 2, the CRDS laser beam propagates horizontally, and the selected particle is confined in horizontal dimensions by either the radial intensity gradients in a vertically upward propagating Bessel laser beam or the potential energy well of the LEQ trap. Vertical positioning is controlled by balancing the radiation pressure force (which scales with the laser power) exerted by the BB or the electrostatic repulsion of an end-cap electrode in the LEQ against the combined forces of the particle weight and a drag force exerted by a gentle downward flow (0.05–0.2 L min^–1^) of humidity-controlled nitrogen gas.^74^ The key advantage of the BB over the more usual optical traps exploited in single-aerosol levitation, formed by tight focusing of a Gaussian laser beam, is that the vertical position of the trapped particle can be manipulated over several millimeters by changing the beam intensity and hence radiation pressure. It also enables isolation of particles over a much broader size range and trapping of smaller particles than the more commonly used gradient force traps. Our designs of BB and LEQ traps are described in greater detail in prior publications.^35−38,40,42,74−77^ Imaging the particle positions in either the BB or LEQ trap shows that the vertical confinement is considerably better than the dimensions of the particle. In our experimental design, most of the particle motion is instead in the horizontal plane, either along or orthogonal to the ring-down cavity axis. In the Bessel beam trap this spatial confinement is to within the core of the Bessel beam (with a radius of <3 μm),^35^ whereas in our first-generation LEQ trap we estimate the particle displacement to be ∼40 μm.

Although confined in three dimensions by the applied forces in the BB or LEQ trap, the particle still undergoes motions that add variance to our measurements of its extinction cross section, even when the particle size and composition can be considered constant. The two main causes of this variance are radial motion that takes the particle away from the center of the Gaussian TEM_00_ mode and motion along the axis of the CRDS laser beam. This latter motion is particularly significant if the particle moves distances comparable to the wavelength of the illuminating light because it then passes through regions of different phases of the standing-wave electromagnetic field confined within the cavity. As Miller and Orr-Ewing showed, the measured extinction depends on where the particle sits within this standing wave structure, with different extinctions when centered at a node or an antinode of the longitudinal cavity mode, or indeed at any phase between these two limits.^78^ Such EM-field phase-dependent extinction behavior deviates from standard Lorenz–Mie theory, which considers the case of plane wave illumination of the particle, but can be accounted for in the analysis of the CRDS data.^35−40,42,78^ Recent simplifications for analyzing single-particle CRDS data are described in Section 3.2.

For a particle trapped at the center axis of a TEM_00_ Gaussian cavity mode, the conversion of experimentally measured ring-down times to extinction cross sections uses eq 2:
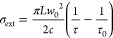
2Here, *L* is the length of the ring-down cavity (defined by the mirror separation), *w*_0_ is the beam waist of the TEM_00_ cavity mode at the position where the aerosol particle is trapped (generally the longitudinal center of the cavity), and *c* is the speed of light in air. The time constants τ and τ_0_ are measured with and without the aerosol particle at the center of the TEM_00_ mode, respectively. This method requires no calibration beyond a baseline measurement of the background ring-down time τ_0_ in the absence of the particle, which is achieved by controllably moving the particle above or below the TEM_00_ mode volume by changing the power in either the BB or the DC electrode voltage of the LEQ trap. The τ_0_ value incorporates the effects of imperfect cavity end-mirror reflectivity (including scattering from the mirror surfaces and diffraction losses) and Rayleigh scattering by gaseous species within the cavity (for our measurements, N_2_) on the decay in intensity of the intracavity laser beam.

**Figure 3:**
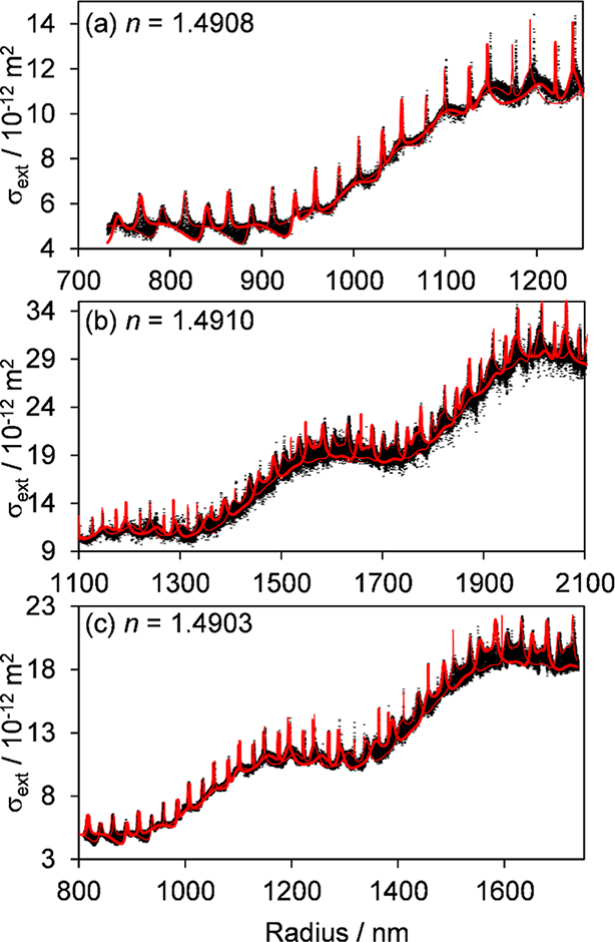
Repeat measurements (black points) of the evolving extinction cross section with particle radius for three separate and nonabsorbing 1,2,6-hexanetriol droplets levitated using a BB optical trap at the center of a 405 nm cw CRDS probe beam. Measurements were performed under dry conditions (RH < 10%). The best fits of the envelopes in extinction predicted by cavity standing wave Mie theory are shown by the red lines; the thick and thin lines represent the predicted cross sections when the particle is located at a node or antinode, respectively, of the intracavity standing wave. The corresponding retrieved real refractive indices at 405 nm from these fits are given in each panel. The measurement and fitting approaches are the same as those reported in ref (37).

A typical measurement on a nonabsorbing droplet isolated using our BB trap proceeds as follows. A single droplet from a nebulizer spray is trapped in the core of a Bessel beam, and its location is manipulated to reach the point of maximum extinction, corresponding to the center axis of the TEM_00_ mode, as determined by the vertical and horizontal positions that give the shortest ring-down time. This careful positioning is achieved through variation in the Bessel beam laser power and translation of the optical trap using a micrometer-precision translation stage. The particle is held in place over extended time scales of minutes to hours using a feedback loop modulating the BB laser power while extinction measurements are made. Illumination of the droplet with a second laser beam, together with camera imaging to measure the spatially varying elastic light scattering distribution (the phase function), provides a first estimate of the evolving droplet radius that is refined in subsequent analysis of the CRDS data. As the droplet size evolves, for example through evaporation of semivolatile components or through hygroscopic response if the ambient RH is controllably altered, the measured extinction sweeps through interference and resonance structure such as is shown in Figure 3. Fitting of the size-dependent extinction cross sections to Lorenz–Mie theory (or modifications thereof to account for the cavity standing wave pattern) gives highly precise and accurate determinations of the droplet refractive index and its variation with droplet size.^39^

A similar procedure is used for absorbing droplets, although the BB trap must be replaced by a LEQ trap that isolates single, charged particles generated using a droplet-on-demand dispenser.^42,79^ The height of the trapped particle is then stabilized by feedback control of the LEQ DC voltage. The following sections provide examples of such measurements for nonabsorbing and absorbing droplets, and they discuss our first efforts to study nonspherical particles. We then highlight work performed elsewhere using a similar methodology to measure the UV–visible extinction properties of nonspherical particles.

### 3.1 CRDS of Single Nonabsorbing Droplets

Our first demonstration of CRDS measurements for single levitated particles exploited a Bessel beam trap, building on our prior experience in the use of these optical traps to manipulate particle positions over macroscopic length scales.^76,77^ Optical trapping of single droplets in a Bessel beam (or in a conventional optical tweezer) precludes study of absorbing droplets because of motion induced by the heating effect of the trapping laser beam. Photophoretic effects arising from the strong temperature gradients across the absorbing particle surfaces caused by high-power-laser irradiation drive particles away from regions of high light intensity and therefore away from the optical trap.^80^ First demonstrations of CRDS measurements of extinction by single droplets therefore concentrated on nonabsorbing particles. A wavelength of 532 nm was chosen for the optical trap, not only because of the ready availability of high-power cw lasers at this wavelength but also because water and other species of interest (e.g., atmospherically relevant inorganic and organic compounds) are only weakly absorbing in this region. Evaporative loss from the trapped droplet (e.g., through the particle-to-gas partitioning of semivolatile compounds or from the loss of water in response to a controlled reduction in ambient RH) results in a steadily decreasing radius and, hence, size parameter. Thus, measurements of extinction recorded over times of minutes or hours will resolve interference and resonance structure in the light scattering cross sections that can be fitted to determine accurately the refractive index of the droplet. For single-component droplets, the real refractive index is invariant with droplet size, whereas for aqueous solutions containing dissolved nonvolatile solutes, the real refractive index evolves as water content is lost.

**Figure 4:**
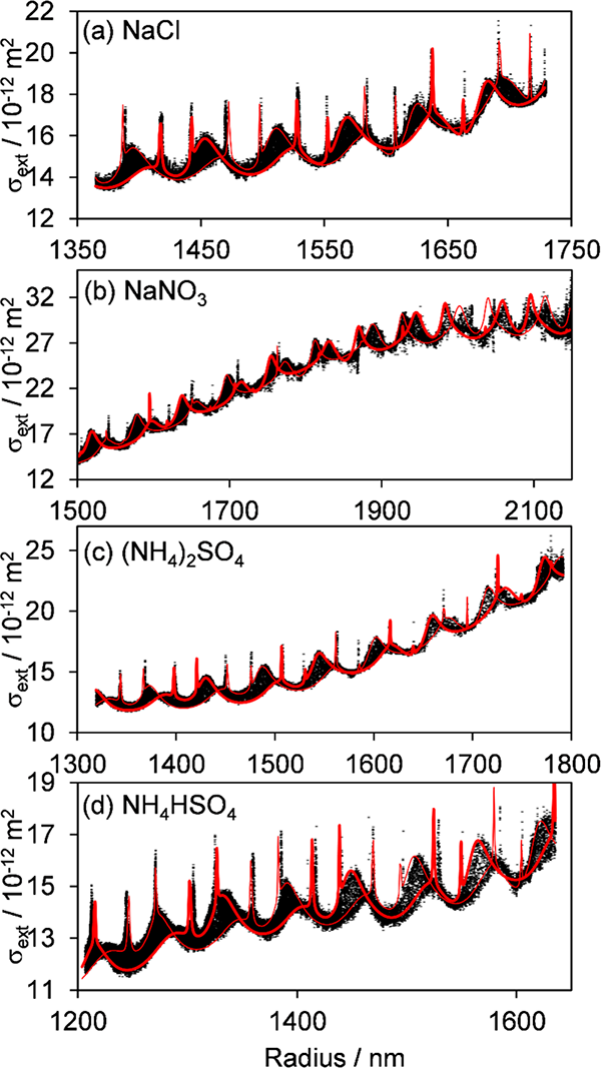
Example measurements (black points) of extinction cross section versus particle size for aqueous droplets containing nonvolatile inorganic solutes. Measurements were performed by levitating droplets using a BB optical trap at the center of our 405 nm CRDS probe beam. During experiments, the relative humidity was reduced steadily over time from a starting RH of ∼85% until the droplet underwent efflorescence (crystallization), driving the continuous evaporation of water. The best fit cavity standing wave Mie envelopes are shown by the red lines, with the thick and thin lines corresponding to node and antinode particle positioning, respectively. The measurement and fitting approaches are the same as those reported in ref (40).

Figures 3 and 4 show examples of CRDS-measured extinction cross sections with evolving particle size (determined from concurrently recorded phase functions) for single-component 1,2,6-hexanetriol droplets and droplets of aqueous solutions of NaCl, NaNO_3_, (NH_4_)_2_SO_4_, and NH_4_HSO_4_. In the experiments with 1,2,6-hexanetriol, the RH was set to dry conditions (RH < 10%) so that droplets with an initial size in the range ∼1.3–2.1 μm evaporated over ∼3–5 h as the semivolatile 1,2,6-hexanetriol partitioned from the droplet into the ambient N_2_ gas stream. The measurement and analysis protocols used for the data sets presented in Figure 3 are described in ref (37). In the experiments with aqueous solutions containing nonvolatile inorganic solutes, shown in Figure 4, droplets were trapped at an initially high RH (∼85%), which was then reduced. With steady water partitioning following the trend in gas phase RH, the solute concentration inside the droplet eventually reached supersaturated concentrations, triggering solute crystallization (efflorescence) to produce a dry particle. For the solutes tested, the effloresced particles were nonspherical and were therefore ejected from the Bessel beam optical trap. Reference (40) provides further details of the methodology for the measurements and the analysis of the data present in Figure 4.

The Mie-scattering structure can clearly be seen in the experimental data, with each measured cross section assigned to a specific droplet radius derived from simultaneous phase function measurements. The variation in the measurements arises from the Brownian motion of the droplet in the core of the BB trap over distances comparable to the wavelength of the probe laser used for CRDS measurements (see the above discussion of intracavity standing wave effects). Fits to extract refractive indices consider the two limiting cases of a droplet located at a node or an antinode of the intracavity standing wave (shown as red curves in the figures), with the refractive index value optimized in fits that maximize the number of experimental measurements falling within this envelope. The other parameters allowed to vary in the fits are the droplet radius (within tight bounds of a few nanometers either side of the value determined from phase function fitting) and the beam waist of the cavity TEM_00_ mode. The latter parameter can be predicted for a cavity of known separation and radius of curvature of the two end mirrors and the wavelength of the light,^81^ but fits are improved by allowing small deviations from these predictions to account for uncertainties in the mirror properties and the horizontal location of the trapped particle along the cavity axis.

In the fits to the data in Figure 4 for hygroscopic response measurements, the relationship between droplet radius *r* and the radius-dependent real part of the refractive index, *n*(*r*), is constrained using
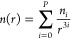
3Here, *n*_0_ is the refractive index of the pure solvent at the probe wavelength and has a known value that is fixed in the fitting (e.g., *n*_0_ = 1.3431 for water at 405 nm at a temperature of 293.15 K). The expansion is generally truncated at *P* = 2. Further details of the fitting procedures are given in our previous publications.^36−40^

Measurements of the type described above provided a comprehensive set of values for the real components of the refractive index at CRDS wavelengths of 405 and 532 nm of aqueous droplets containing various inorganic salts found in atmospheric aerosol particles.^40^ In addition, the wavelength dispersions of these refractive indices were quantified by refractive index retrievals from phase function measurements with laser excitation at 633 and 473 nm. In combination with our CRDS measurements, these data provided full refractive index parametrizations for various inorganic solute droplets over the full visible spectrum (λ = 400–700 nm) and with variation in RH across the full range over which the deliquescent droplets exist. These data are now tabulated in the University of Oxford Aerosol Refractive Index Archive (ARIA, http://eodg.atm.ox.ac.uk/ARIA/data) for use by atmospheric modellers in radiative forcing calculations.

### 3.2 CRDS of Absorbing Droplets

For accurate and sensitive extinction cross-section measurements to be made on light-absorbing droplets and for the associated complex refractive index to be retrieved accurately, a step-change is needed in our measurement approach. As was noted earlier, transitioning from a BB optical trap to a LEQ trap allows such CRDS measurements to be made on single, absorbing particles or droplets from which values for both the real and imaginary components of the refractive index can be extracted. The approach is otherwise similar to that for nonabsorbing particles and exploits the continuous change in radius of a droplet driven by either the loss of one component by evaporation in a low-RH environment or the hygroscopic response of the droplet to changes in RH. Very recently, we have demonstrated a simplification in the analysis of such data that is particularly beneficial for such measurements made on absorbing particles. We average the single-particle extinction data points from a nominal CRDS measurement sampling rate of ∼20 Hz to a reduced sampling rate of 1 Hz, giving cross sections corresponding to those predicted by Lorenz–Mie theory because the effects of the standing wave are removed by particle motion.^79^ The averaged data can therefore be fitted directly to a Lorenz–Mie theory model to extract refractive index values.

For a droplet continuously losing mass by evaporation, CRDS measurements map out the changes to the interference and resonance structure in the optical extinction, and we have recently shown that the radius-dependent data can be fitted to obtain values of *n* and *k*.^79^ This fitting requires parametrization of the size dependence of these two components of the complex refractive index. The real component at a particular wavelength is expressed as the expansion in droplet radius given in eq 3. Meanwhile, it can be shown from first-principles that the imaginary component of the complex refractive index for a mixture of a nonabsorbing semivolatile species with a light absorbing involatile species is described in terms of the droplet radius by
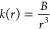
4with the parameter *B* obtained from the fits. Application of eqs 3 and 4 to constrain the size-dependent real and imaginary components of the refractive index for mixed-composition droplets is valid if the droplets are not undergoing chemical or physical changes other than evaporative loss or condensational gain of a volatile component. However, droplets containing involatile chromophores that are susceptible to photobleaching or that undergo chemical transformations will require explicit consideration of the variation of *n* and *k* with time. Exploring these more complicated parametrizations will be a next step in the development of our methods.

**Figure 5:**
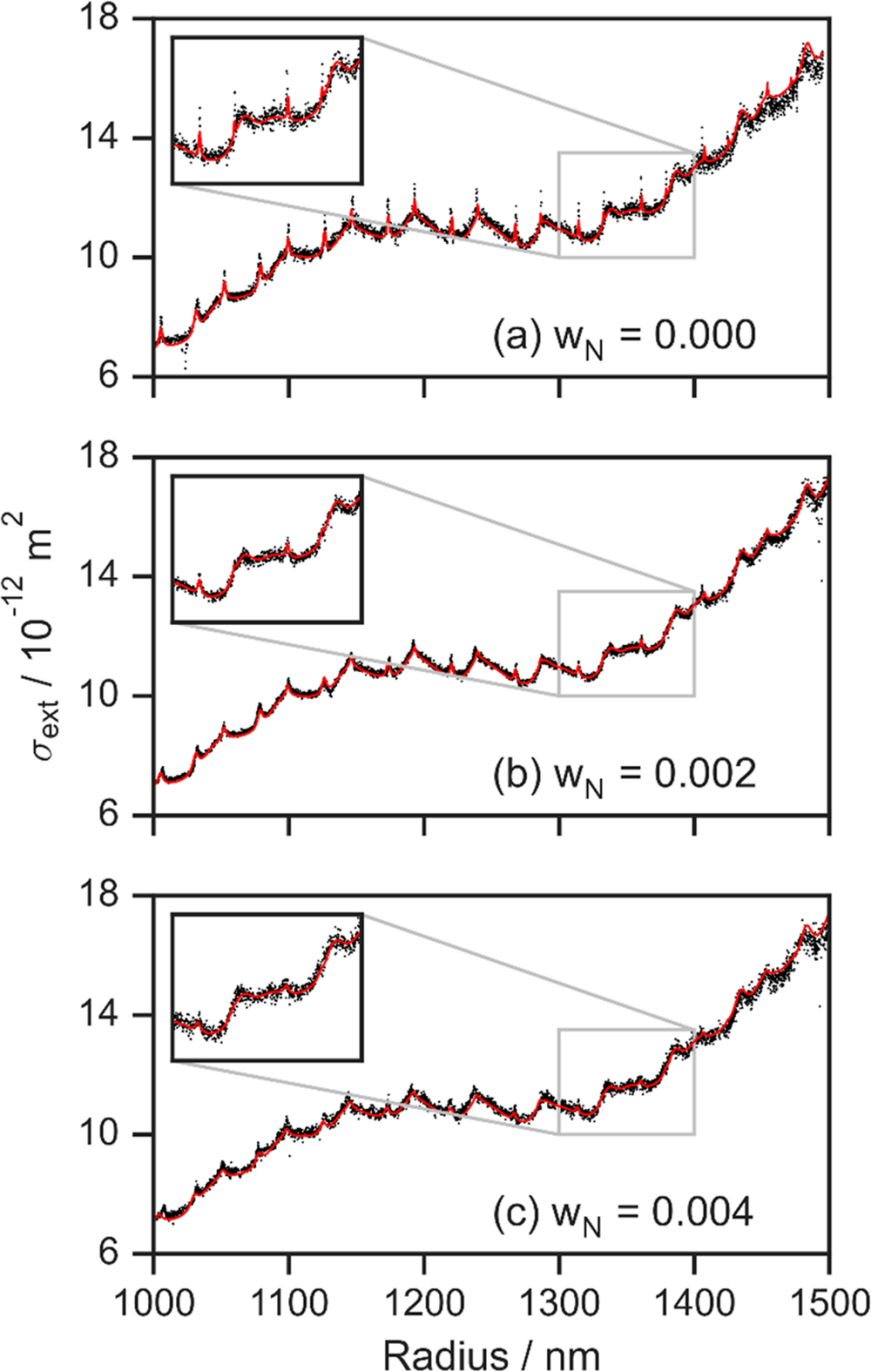
Measured dependence of the extinction cross section on particle radius (black points) for droplets with different absorption strengths, determined by the mass fraction (*w*_N_) of nigrosin dye (a nonvolatile component) internally mixed with 1,2,6-hexanetriol. Droplets were levitated using our LEQ trap and positioned at the center of a 405 nm CRDS probe beam. The initial mass fraction of nigrosin dye in the binary droplet is indicated in each panel. The corresponding best fits to Mie theory extinction calculations are also shown (red lines) and allow retrieval of the real and complex components of the refractive index at 405 nm. The measurement and fitting approaches are described in ref (79). Reprinted with permission from ref (79). Copyright 2022 American Chemical Society.

Although it is generally challenging to separate scattering and absorption losses, our approach is successful because absorbing spherical droplets have lower cavity *Q*-factors for their whispering gallery modes than their nonabsorbing counterparts. Consequently, the resonance structures in the extinction cross sections corresponding to WGMs are broader and of lower amplitude for the absorbing droplets.^66^ Nevertheless, the broad interference structure persists sufficiently (see Figure 1c) to be exploited in the fits. Examples of fits of Mie theory calculated σ_ext_ values to measurements for droplets composed of a binary mixture of nigrosin dye (typically with an initial mass concentration of 0.2–0.4%) with 1,2,6-hexanetriol are compared with the corresponding measurements for a pure 1,2,6-hexanetriol droplet in Figure 5. Nigrosin dye is nonvolatile, and therefore the particle size and complex refractive index of the droplets evolve as the nonabsorbing 1,2,6-hexanetriol component evaporates steadily in the low-humidity (RH < 10%) environment. The progressive dampening of the interference and resonance structures as the initial mass fraction of nigrosin dye increases is clear from this figure.

**Figure 6:**
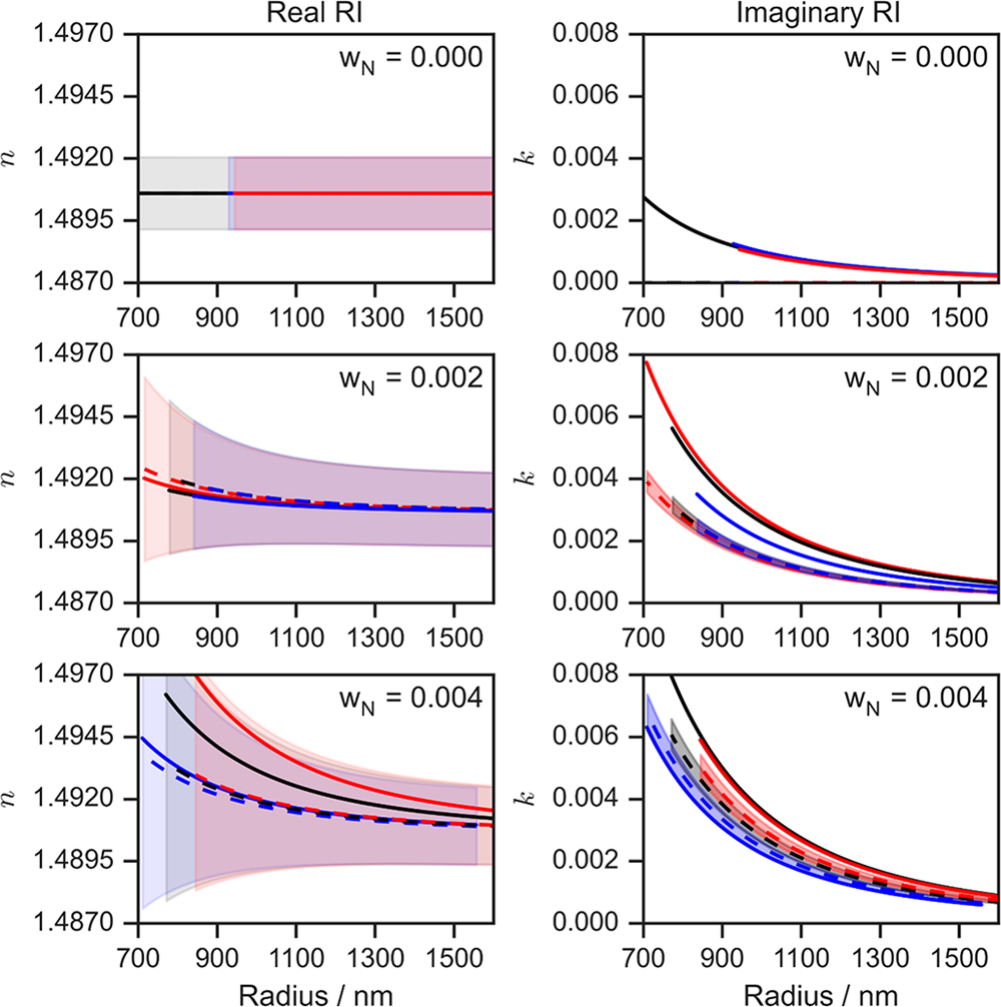
Retrieved (solid lines) and predicted (dashed lines) refractive indices versus particle radius for droplets with different absorption strengths, determined by the initial nigrosin mass fraction of nigrosin dye (*w*_N_) internally mixed with 1,2,6-hexanetriol. The compositions presented are *w*_N_ = 0.000 (top panels), 0.002 (middle panels), and 0.004 (bottom panels). The shaded envelopes represent the uncertainties in the predicted *n* and *k* values as a function of particle size. The three different colored lines on each panel correspond to the three repeat droplets that were studied for each initial droplet w_N_ value. Reprinted with permission from ref (79). Copyright 2022 American Chemical Society.

Fits of Lorenz–Mie theory to the full set of extinction data in Figure 5 for the absorbing droplets retrieve the size-dependent real and imaginary components of the refractive index shown in Figure 6. The values are in accord with mixing rule predictions calculated from the known refractive indices of nigrosin and 1,2,6-hexanetriol at 405 nm;^79^ the variation in *n* with particle size is predicted using a mole fraction weighting of molar refraction,^82^ and predictions for *k* use a mass fraction weighting of the pure component imaginary refractive indices. These mixing rules for *n* and *k* are chosen because of their underlying physical basis. The so-called molar refraction mixing rule is self-consistent with the Lorentz–Lorenz model describing the manifestation of *n* (a macroscopic quantity) from an atomistic description of matter, while the mass fraction mixing rule for *k* accords with the scaling of light absorption in proportion to the concentration of chromophores in dilute mixtures (i.e., as described by the Beer–Lambert law). Figures 5 and 6 show that we resolve sensitively the impacts on the extinction cross sections of small increases in the initial chromophore mass concentration corresponding to 0.2% of the total droplet mass, and the resolved changes in *k* are far below typical ranges of *k* for absorbing atmospheric aerosol particles (with soot and BrC having *k* in the visible spectrum of ∼0.8 and ∼10^–3^ to 10^–1^, respectively).

Although the precision of these determinations of *n* and *k* values for single, micron-scale droplets is high, we recognize that our approach has some limitations. The upper size limit for light-absorbing droplets studied with our current instrument design is a diameter of ∼4 μm. For larger particles, the attenuation of the intracavity beam intensity and the shortening of the cavity ring-down time prevent CRDS measurement of extinction cross sections with the precision required for complex refractive index retrievals. We are in the process of exploring several strategies to extend this upper size limit. For the range of *n* values expected for aqueous droplets containing organic or inorganic solutes of importance to atmospheric chemistry, we do not anticipate any limitations to reliable experimental determinations.^40^

So far, the method has only been demonstrated for weakly absorbing particles (with *k* ≤ 0.02 at the phase function imaging wavelength of 532 nm), in part because our phase function images for particles with higher absorption strengths do not retain sufficient contrast in the interference structure for initial size estimations with suitable accuracy. In addition, the signal-to-noise ratios in the recorded phase functions degrade as increasing particle absorption reduces the amount of elastic light scattering. Imaging this elastic light scattering to locate the levitated particle’s position is also necessary to maintain the particle at the center of the cavity TEM_00_ mode. As the particles become more absorbing, the WGM resonance structure in the Lorenz–Mie scattering, which is exploited in our CRDS data fitting, broadens and reduces in amplitude. However, our expectation is that the broader interference structure included in the fits of measured cross-section values will remain informative, and hence that our CRDS measurements can still be used to extract higher *k* values, provided we can accurately determine the particle size and keep it stably levitated in the center of the ring-down cavity. Regardless of the above limitations, the accessible size and absorption windows offer scope for wide-ranging measurements on particles of atmospheric importance, such as brown carbon. Excluding mineral dusts and cloud droplets, atmospheric aerosols are overwhelmingly measured to have particles diameters below 1 μm.^83^ Such sizes are compatible with our demonstrated cross-section measurements on particles with radii as small as ∼300 nm.^37^

### 3.3 CRDS for Nonspherical Particles

In addition to the benefits of the LEQ trap described in Section 3.2 for study of the optical properties of absorbing droplets, it also offers a new capability for quantitative measurements of the extinction cross sections for nonspherical particles. As we recently demonstrated, particles initially trapped as spherical, aqueous droplets remain confined in the trap after efflorescence, even if the resulting solid particle is nonspherical.^42^ Continuous extinction measurements were made on aqueous NaCl or (NH_4_)_2_SO_4_ droplets as the ambient RH was reduced progressively until the droplets effloresced to produce solid particles. In each case, the efflorescence point was clear from a discontinuous change in extinction cross sections, followed by no further evolution in the mean extinction because the dry particles underwent no additional loss of mass. Interpretation of the extinction cross sections for the dry particles of uncertain shape remains a challenge, with first attempts made using the T-matrix/Extended Boundary Condition Method (EBCM) for random orientations of the particle within the probe light field and spheroid or superellipsoid parametrizations to infer information about the particle shapes. Notably, our model-to-measurement comparisons showed that the extinctions of the formed (NH_4_)_2_SO_4_ crystals were accounted for by Mie theory, while those for NaCl could only be reconciled by models including nonsphericity.

Complementing these applications of our single-particle CRDS methods, Wang and co-workers have developed similar instruments to study nonspherical aerosol particles.^84,85^ Their first measurements used two loosely focused, counter-propagating Gaussian laser beams (λ = 405 nm) to form a radiation pressure trap.^85^ Stable trapping was demonstrated for single particles consisting of either single-wall or multiwall carbon nanotubes with sizes in the range 9–60 μm. These levitated particles were then manipulated into the center of a pulsed CRDS spectrometer (pulse duration of 10 ns, repetition rate of 20 Hz) for which the wavelength could be scanned over the range 315–320 nm. In this way, single-particle extinction was measured for the nanotube systems of interest. Further development of their instrument to include a focused hollow-beam optical trap enabled measurement of the single-particle extinction by various mineral dust particles with sizes in the range 16–32 μm, including mined minerals from the San Francisco volcano field and two types of volcanic ash (from Puú Nene in Hawaii and Eyjafjallajökull in Iceland), or light absorbing carbon spheres with diameters in the range 2–4 μm.^84^ In their design, fine-tuning of the spatial overlap of incident and retro-reflected hollow beams created an *optical bottle*, comprising an axisymmetric dark void surrounded by regions of high laser intensity. This optical bottle confines absorbing particles using repulsive photophoretic forces in the bright regions.^80^ This novel optical trapping approach allowed stable trapping and fine manipulation of solid particles with varying shapes and absorption strengths and subsequent measurements of single-particle extinctions at λ = 308 nm. However, quantitative information was not extracted about the particle microphysics (e.g., complex refractive index or morphology), and the data were not used to test electromagnetic models of light-particle interactions.

## 4 Summary and Outlook

In this Feature Article, we have underscored the unique ability of single-particle CRDS to provide extinction cross-section measurements to a level of sensitivity and accuracy that enables the robust retrieval of aerosol physicochemical information, including complex refractive index and particle morphology. This technique has been applied to different particle types found in the atmosphere, including spherical and nonspherical particles with a range of absorption strengths. It now offers opportunities to address several outstanding challenges in atmospheric chemistry. One such challenge motivating our current research is to understand and quantify the optical properties of organic aerosols absorbing strongly at short visible wavelengths (so-called brown carbon, BrC).^86^ Numerous organic species absorb in the visible window, complicating the tractability of this problem. Moreover, these species undergo photooxidation chemistry that bleaches the chromophores and therefore drives temporal evolution of the light absorption over the atmospheric lifetimes of aerosol particles.^8,87^ Measuring the complex refractive indices for droplets containing various BrC chromophores is a challenge well-suited to our single-particle CRDS technique. Deriving the changes in *n* and *k* as levitated organic particles are exposed to reactive gases (e.g., NO_*x*_, O_3_) and actinic flux will deepen current understanding of the kinetics of chromophore formation and loss in the natural environment. Better characterization of BrC optical properties and their temporal dependence under atmospheric conditions will contribute to future improvements in climate models. Indeed, the aforementioned complexities in attributing and quantifying BrC absorption mean that climate models commonly neglect these contributions and instead focus on black carbon, mineral dust, and sulfate aerosols,^88^ even though BrC absorbs significantly in the near-UV and short-wavelength visible regions.

The constituent components of aerosol particles rarely exist as separate entities but are instead internally mixed, with any given particle containing multiple species with complex mixing states such as coated BC or phase-separated organic–inorganic particles.^89,90^ Using single-particle CRDS measurements to challenge existing optical models and parametrizations to treat such mixed systems could help reconcile active debates over the best model representations. Similarly, our techniques could test models for the optical properties of nonspherical mineral dust aerosols and how these properties evolve with hygroscopic growth. At high altitudes in the free troposphere, the ice particles constituting cirrus clouds adopt a wide range of fractal geometries that scatter light in complex ways, and much research in recent years has focused on representing these properties in radiative transfer models.^91^ Not only do ice particles scatter visible light strongly, but they also have near- and mid-IR absorption bands and can thus contribute a significant net positive radiative forcing by trapping outgoing terrestrial radiation. Extending our measurement capability to the IR region, or indeed to short UV wavelengths with likely relevance to photobleaching phenomena, should therefore offer new research avenues with potential for high impact.

More broadly, we propose that our approach based on single-particle CRDS is a powerful platform for studying the formation and loss of absorbing species important in other fields of aerosol science. For example, we expect future applications will enable better-constrained estimates for the kinetics of chemical reactions accelerated in micron-scale droplets,^16−18^ from which much can be learned about the influence of unique aerosol physicochemical properties. These include the high surface area to volume ratios of small droplets (which will enhance the role of heterogeneous chemistry),^15^ reactions in solution under supersaturated conditions, strong gradients in pH between the droplet bulk and particle surface, and size-dependent internal electromagnetic fields that can govern the rates of photochemical reactions.^50^

Addressing the above problems in atmospheric and aerosol science relies on further improvements to the sensitivity of our measurements and careful assessment of the limitations of our data. We have previously published comprehensive analyses of complex refractive index retrieval accuracies from extinction measurements made using our 532 nm CRDS instrument, demonstrating that *n* is retrieved to better that 0.02% and *k* is retrieved to an accuracy in the range 1–20% depending on the magnitude of *k*.^39^ However, such assessments require continuous re-evaluation as we pursue ever-improved sensitivities in our extinction data, extend measurements to other wavelength regions and to particles of varying absorption strengths, and develop new data analysis approaches for the retrieval of aerosol physicochemical information. Indeed, a forthcoming publication will describe our latest improvements to our measurement and retrieval approaches, and it will provide estimates of the accuracies in retrieved *n* and *k* for our latest 405 nm CRDS measurements on light absorbing droplets isolated in our LEQ trap. This assessment includes consideration of the amplitudes of particle motion away from the center of the TEM_00_ cavity mode, which introduces systematic errors into the determination of σ_ext_ values. In the BB optical trap, the particle motion is Brownian and is restricted to within the <3 μm core radius of the BB, which is much smaller than the ∼260 μm beam waist of the TEM_00_ mode. At the current performance levels of our CRD spectrometer, the resulting errors are negligible. In contrast, particle motion in the LEQ trap is driven by the harmonic AC electric fields applied to the quadrupole electrodes. In our current LEQ trap, this driven motion has an estimated amplitude of ∼40 μm that is sufficient to introduce a small but detectable bias in the measured ring-down times, which can be compensated to some degree in our fitting procedures. Refinements to the design of the LEQ trap to confine the particle motion more tightly will improve the accuracy of our future measurements of the complex refractive indices of a range of absorbing aerosol particles.
